# Case Report: Abnormal uterine bleeding caused by displacement of an intrauterine device

**DOI:** 10.3389/fmed.2026.1765829

**Published:** 2026-03-04

**Authors:** Zhixiang Zou, Yijia Du, Jianjian Wang, Bifeng Fu

**Affiliations:** 1 Department of Gynecology, The First Hospital of Hunan University of Chinese Medicine, Changsha, Hunan, China; 2 Department of Traditional Chinese Orthopedics and Traumatology Ward, National Clinical Research Center for Chinese Medicine Acupuncture and Moxibustion, The First Teaching Hospital of Tianjin University of Traditional Chinese Medicine, Tianjin, China

**Keywords:** abnormal uterine bleeding, case report, IUD displacement, surgery, uterine perforation

## Abstract

Though rare, complications associated with intrauterine devices (IUDs) can present with atypical symptoms, leading to diagnostic challenges. This case report describes a 36-year-old multiparous woman with a history of IUD insertion who presented with intermittent light bleeding menstrual periods for 1 year, in the without abdominal pain or dysmenorrhea. Imaging studies (including pelvic X-ray and ultrasound) revealed complete uterine perforation and IUD displacement into the pelvic cavity. The IUD was successfully removed through a combined hysteroscopic and laparoscopic procedure. This case emphasizes that severe complications, such as IUD perforation and displacement, may manifest solely as minor abnormal uterine bleeding. This finding suggests that even in the absence of pain, patients with persistent irregular bleeding patterns should remain highly vigilant for IUD-related complications. Since ultrasonography remains the gold standard for assessment, timely diagnosis of these complications is crucial to prevent potential long-term sequelae.

## 1 Introduction

The intrauterine device (IUDs) are a commonly used, effective, and safe long-acting contraceptive method. About 14% of people worldwide use IUDs, which amounts to approximately 250 million users. In China, about 41% of women of reproductive age use IUDs (approximately 141 million users), accounting for over 50% of all contraceptive methods in the country (1). However, IUDs can lead to various complications, including displacement, embedment, perforation, fragmentation, and contraceptive failure (2). The clinical symptoms of IUD complications are diverse, primarily manifesting as pain, abnormal menstrual volume, or intermittent bleeding (3). When clinical symptoms are atypical, the initial clinical diagnosis of IUD complications can be challenging. Therefore, this article reports a case of complete displaced IUD with perforation, presenting only as slight intermittent vaginal bleeding and without significant abdominal pain.

## 2 Case presentation

**Figure 1:**
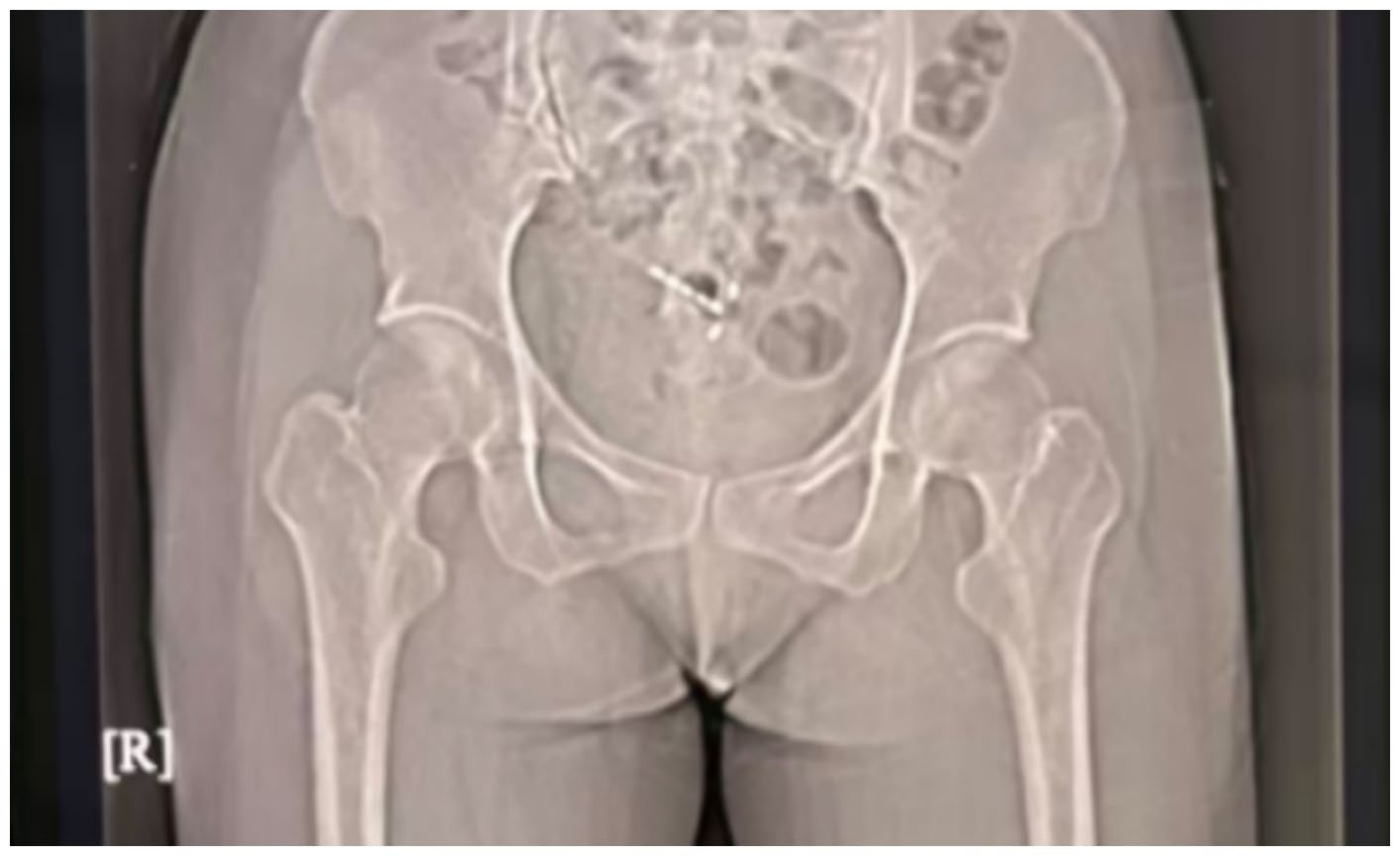
Preoperative pelvic X-ray image.

**Figure 2:**
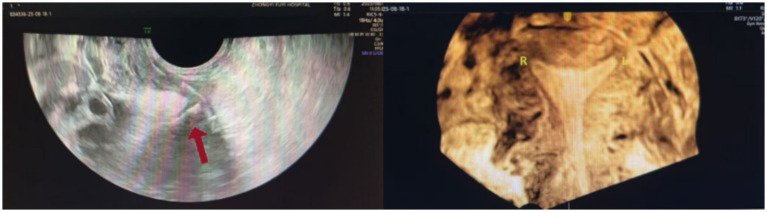
Preoperative ultrasound image.

**Figure 3:**
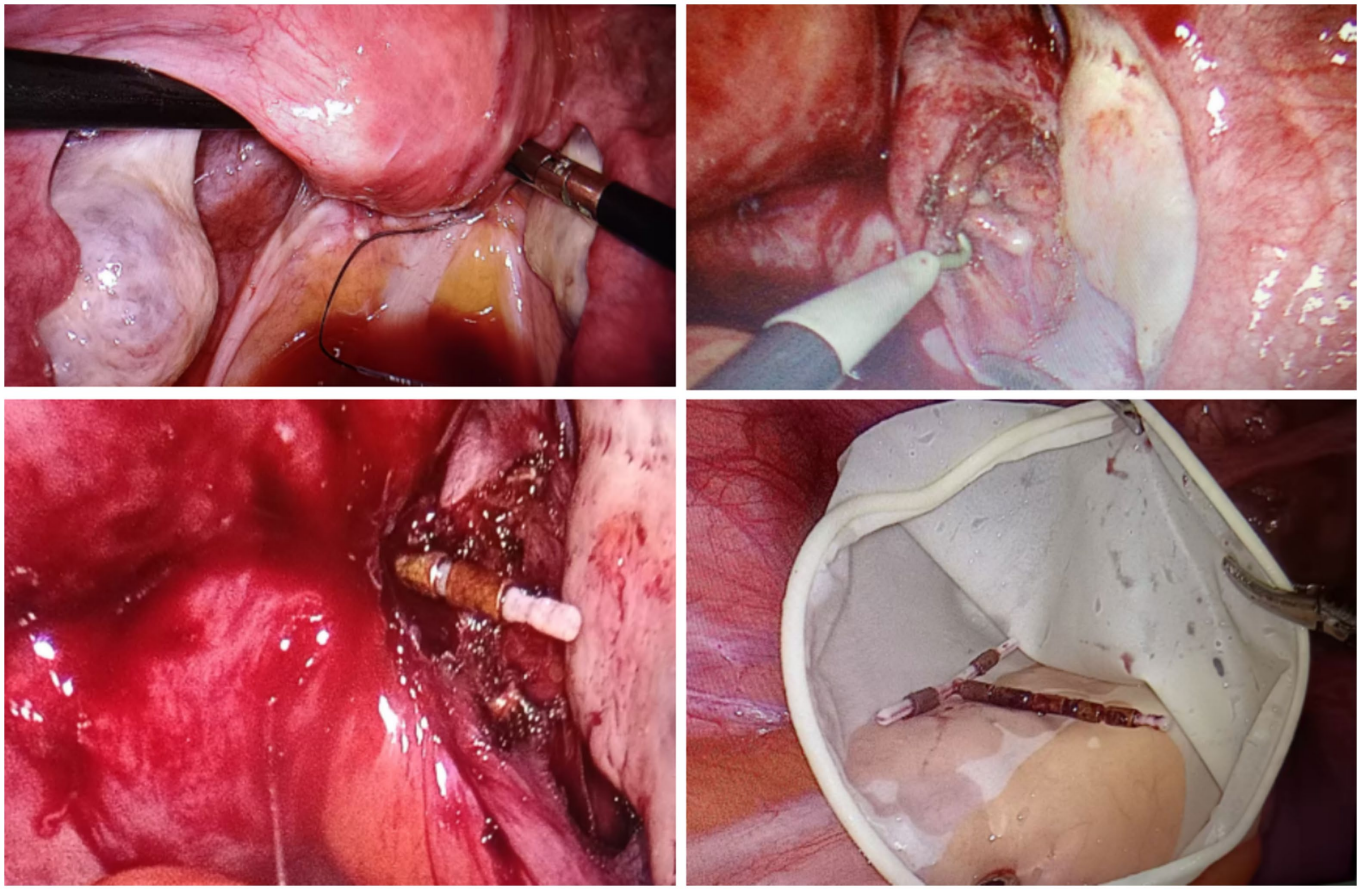
Laparoscopic surgical images (intraoperatively, the tail thread of the IUD was visualized in the rectouterine pouch, while an IUD-like foreign body was located in the posterior right wall of the cervix, within the area of adhesions involving the pelvic wall and intestines. Using an ultrasonic scalpel and a monopolar electrode, adhesions were gradually dissected, and a “T-shaped” intrauterine device was completely removed).

A 36-year-old woman (7 pregnancies, 2 births) presented to our gynecology department with a complaint of recurrent menstrual bleeding lasting 1 year. The patient had an IUD inserted in 2020 and had not undergone any follow-ups until presentation. In March 2024, she underwent cervical conization due to cervical intraepithelial neoplasia (CIN III). Postoperative menstrual bleeding continued until the onset of the next menstrual cycle; this bleeding was light brown, lasted longer than usual, and was not accompanied by abdominal pain or dysmenorrhea. A pelvic X-ray (Figure 1) examination suggested that the IUD was malpositioned. Ultrasound (Figure 2) further confirmed the diagnosis: showing no clear IUD echo within the uterine cavity. A roughly “T-shaped” IUD echo was primarily located outside the uterine contour. The lower segment of the IUD protruded from the posterior fornix of the cervix on the right side, extending obliquely and closely adhering to the right uterine wall. The patient underwent hysteroscopic exploration and laparoscopic IUD removal. Hysteroscopic examination revealed no IUD within the uterine cavity, and a “T-shaped” IUD was ultimately removed from the abdominal cavity via laparoscopy (Figure 3).

## 3 Discussion

As an effective contraceptive tool, IUDs can significantly reduces the incidence of unintended pregnancy, short-interval pregnancy, and induced abortion, and are widely used globally. Problems related to IUD insertion are relatively rare, with the incidence of uterine perforation being particularly low. A multicenter cohort study involving 326,658 subjects indicated that the cumulative incidence of uterine perforation was as low as 0.21% after 1 year and 0.61% after 5 years (4). Due to the extremely incidence of uterine perforation and atypical clinical symptoms in some patients, misdiagnosis can easily occur in clinical practice. Some patients may only discover perforation when they present with abdominal pain, cramping symptoms, or during imaging evaluation prior to requesting removal of the IUD (4); therefore, prompt imaging is crucial.

Imaging assessment of IUD displacement or perforation can combine various examination methods. X-ray examination has limitations in accurately locating the IUD position and cannot effectively distinguish whether the IUD is within the uterine cavity or has perforated and displaced into the abdominal or pelvic cavity (5). Computed tomography (CT) and magnetic resonance imaging (MRI) are not typically used as routine methods for IUD assessment, but imaging signs of IUD displacement may be incidentally discovered during examinations for other indications. Ultrasound imaging is the preferred method for assessing IUD position and related complications (4). It effectively identifies low-lying IUDs, associated infectious lesions, myometrial embeddings (i.e., partial embeddings of the IUD into the uterine myometrium), uterine perforation, IUD-related intrauterine or ectopic pregnancy, as well as retained or fragmented IUDs. Three-dimensional ultrasound can obtain three-dimensional imaging data of the entire uterus, and reconstruct coronal images of the endometrial cavity. This perspective clearly displays the overall morphology of the IUD (including the stem and both arms) and its spatial relationship with the uterine cavity; it allows for accurate determination of whether the IUD is located within the uterine cavity and particularly aids in identification of abnormal positions (such as arm embedment in the myometrium) (6).

This case highlights the atypical presentation of IUD complications, as the only symptom in the patient was slight abnormal uterine bleeding with no typical abdominal pain, yet her IUD had completely perforated and displaced to an ectopic location in the pelvic cavity. Therefore, for patients experiencing persistent abnormal bleeding after IUD insertion, even in the absence of abdominal pain, the possibility of IUD complications must be highly suspected. Ultrasound examination (US) is the preferred imaging modality for assessing potential IUD complications. Timely imaging assessment and hysteroscopy examination help clarify the diagnosis and avoid missed diagnoses.

## 4 Risk factor analysis

The patient in this case was a multiparous woman (gravidity 7, parity 2), and had a history of cervical conization, which may increase the risk of IUD malposition. Multiple pregnancies may lead to changes in uterine morphology and/or myometrial structure. Additionally, cervical surgery may affect the uterine environment and compromise IUD stability. Therefore, clinicians should closely monitor IUD positions in patients with similar medical histories.

## 5 Limitations of the case

This is a single case report, and while it serves as a warning, it cannot be used to infer the general clinical manifestations of IUD perforation. Future prospective studies are needed to clarify the risk factors and the natural course—including progression, resolution, and potential complications—of asymptomatic IUD malposition.

## Data Availability

The original contributions presented in the study are included in the article/supplementary material, further inquiries can be directed to the corresponding authors.
